# Description of two novel species, *Flavobacterium suaedicola* sp. nov. and *Flavobacterium wonjuense* sp. nov.

**DOI:** 10.71150/jm.2607006

**Published:** 2026-10-31

**Authors:** Seung Yeol Shin, Heeyoung Kang, Yihyun Jeon, Song-Ih Han, Ahyoung Choi, Jaeho Song

**Affiliations:** 1 Division of Biodiversity Inventory, Honam National Institute of Biological Resources, Mokpo 58762, Republic of Korea; 2 Department of Microbial Biotechnology of Science & Technology, Mokwon University, Daejeon 35349, Republic of Korea; 3 Division of Biodiversity Conservation, Honam National Institute of Biological Resources, Mokpo 58762, Republic of Korea; 4 Division of Taxonomy, Honam National Institute of Biological Resources, Mokpo 58762, Republic of Korea; 5 Biological Resources Research Department, Nakdonggang National Institute of Biological Resources (NNIBR), Sangju 37242, Republic of Korea

**Keywords:** *Flavobacterium*, novel species, halophyte, soil

## Abstract

Two novel bacterial strains, designated HNIBRBA15423^T^ and 25HG05S-40^T^, were isolated from the root of a halophyte and from a riverbank soil in the Republic of Korea, respectively. Cells of both strains were Gram-stain-negative, yellow-pigmented, non-motile, and rod-shaped. Phylogenetic analysis based on 16S rRNA gene sequences revealed that both strains belong to the genus *Flavobacterium*. Strains HNIBRBA15423^T^ and 25HG05S-40^T^ showed the highest sequence similarity to *Flavobacterium jejuense* EC11^T^ (98.5%) and *Flavobacterium dankookense* ARSA-19^T^ (98.3%), respectively. The major cellular fatty acids of both strains included iso-C_15:0_, iso-C_15:1_ G, and iso-C_17:0_ 3-OH. Both strains contained menaquinone-6 as the respiratory quinone and phosphatidylethanolamine as the major polar lipid. The complete genome sizes and DNA G + C contents were 4.29 Mb and 29.7% for strain HNIBRBA15423^T^, and 3.45 Mb and 36.6% for strain 25HG05S-40^T^. The average nucleotide identity (ANI) and digital DNA-DNA hybridization (dDDH) values were calculated. For HNIBRBA15423^T^, ANI value compared to *F. jejuense* EC11^T^ were 85.2% with dDDH value of 29.5%. For 25HG05S-40^T^, ANI values compared to *F. dankookense* DSM 25687^T^ and *F. wongokense* WG47^T^ were 76.0% and 78.6%, respectively, with dDDH value of 20.3% and 21.5%. Based on the polyphasic taxonomic data, strains HNIBRBA15423^T^ and 25HG05S-40^T^ represent two novel species within the genus *Flavobacterium*, for which the names *Flavobacterium suaedicola* sp. nov. and *Flavobacterium wonjuense* sp. nov. are proposed.

## Introduction

The genus *Flavobacterium*, a member of the family *Flavobacteriaceae* within the phylum *Bacteroidota*, was first proposed by Bergey et al. (1923) and its description has subsequently been emended (Bernardet et al., 1996; Dong et al., 2013; Kang et al., 2013; Kuo et al., 2013). Since the first species, *Flavobacterium aquatile*, members of the genus have been found in a wide range of ecological habitats, including freshwater (Jo et al., 2023), seawater (Kitahara et al., 2023), algae (Park et al., 2015), soil (Yoon et al., 2006), rhizosphere (Kämpfer et al., 2025), and freshwater fish (Kämpfer et al., 2020). Most species of the genus *Flavobacterium* are Gram-negative, aerobic, rod-shaped bacteria that lack flagella and exhibit are yellow to orange color due to the presence of flexirubin-type pigments or carotenoids. Menaquinone-6 (MK-6) as the predominant respiratory quinone and phosphatidylethanolamine as major polar lipids were typically found in *Flavobacterium* species (Bernardet and Bowman, 2006; Bernardet et al., 2002). Regarding genomic characteristics, the genomes of the genus *Flavobacterium* range in size from 2.3 to 6.1 Mb, with a DNA G + C content of 29.4 to 50.9% (Kim et al., 2023). Metabolic analysis of *Flavobacterium* genomes revealed a wide range of enzymes, including agarase, cellulase, chitinase, chitosanase, dextranase, hyaluronidase, lysozyme, xylanase, and *α*-glucosidase (Jo et al., 2023). The genus is well known not only for its wide distribution, but also for its ecological role in degrading a wide range of high-molecular-weight organic compounds and complex polysaccharides in diverse environments (Lian et al., 2025; Yang et al., 2025).

As of July 2026, the genus *Flavobacterium* comprises 412 species validly published species according to the List of Prokaryotic names with Standing in Nomenclature (LPSN) (Parte et al., 2020). Among *Flavobacterium* species, 112 species have been isolated from diverse habitats in the Republic of Korea. In the present study, during our ongoing survey of indigenous bacterial diversity in Korea, we obtained two novel bacterial strains, designated HNIBRBA15423^T^ and 25HG05S-40^T^, from the root of the halophyte *Suaeda maritima* and riverbank soil, respectively. In this study, whole-genome analysis of strains HNIBRBA15423^T^ and 25HG05S-40^T^ revealed complete circular genomes of 4.29 Mb and 3.45 Mb, respectively. Based on these polyphasic taxonomic and genomic distinctions, we propose that these strains represent two novel species of the genus *Flavobacterium*.

## Materials and Methods

### Isolation and strains

Strain HNIBRBA15423^T^ was isolated from the root of halophyte *Suaeda maritima* collected in Taean, the Republic of Korea (36°49′49.9′′ N, 126°10′48.2′′ E) in June 2024. The root sample was gently cleansed with sterilized phosphate buffered saline (PBS) solution and subsequently homogenized by a sterilized pestle. Supernatant of the homogenized sample was inoculated on marine agar 2216 (MA; BD Diagnostics) and incubated at 20°C for seven days. Although initially isolated on MA, strain HNIBRBA15423^T^ was found to grow well on R2A agar, which was subsequently used for routine culture and characterization to ensure consistent testing conditions with other reference strains. Strain 25HG05S-40^T^ was isolated from a riverbank soil sample collected from Wonju, the Republic of Korea (37°23′7.8′′ N, 127°59′10.4′′ E) in December 2024. The soil sample was serially diluted with sterilized PBS solution. Aliquots of the serially diluted solution were plated onto R2A agar (BD Diagnostics) and incubated at 20°C for seven days. Two distinct yellow-pigmented colonies, designated HNIBRBA15423^T^ and 25HG05S-40^T^, were selected, purified by repeated streaking on R2A agar, and routinely cultured at 25°C. For long-term preservation, the purified strains were stored as glycerol suspensions (20%, v/v) at -80°C. For phenotypic and chemotaxonomic comparisons, closely related type strains were selected based on 16S rRNA gene sequence similarities (*Flavobacterium jejuense* KCTC 42149^T^, *Flavobacterium dankookense* KCTC 23179^T^, and *Flavobacterium profundi* KACC 18559^T^) and inclusion of the type species of the genus (*Flavobacterium aquatile* KACC 11692^T^). These type strains were obtained from the Korean Collection for Type Cultures (KCTC) and Korean Agricultural Culture Collection (KACC), respectively, and cultured on R2A agar at 25°C alongside the novel strains under identical conditions.

### 16S rRNA gene phylogeny

Genomic DNA of strain HNIBRBA15423^T^ and 25HG05S-40^T^ were extracted using the DNeasy Blood and Tissue kit (Qiagen) according to the manufacturer’s instructions. The 16S rRNA genes of the strains were amplified by PCR using universal primers 27F and 1492R (Weisburg et al., 1991) and sequenced by a Sanger sequencer (Macrogen) and subsequently identified using the 16S-based ID application in the EzBioCloud (Chalita et al., 2024). To clarify the phylogenetic position, MEGA version 11 (Tamura et al., 2021) was employed to generate phylogenetic trees based on 16S rRNA gene sequences aligned with the ClustalW (v1.83) integrated in to MEGA11 (Thompson et al., 1994). The resultant phylogenetic trees were constructed based on maximum-likelihood method (Felsenstein, 1981) with the Tamura-Nei model, neighbor-joining method (Saitou and Nei, 1987) with the Kimura-2 model, and minimum-evolution method (Fitch, 1971) with Jukes-Cantor correction. Robustness of the trees was ensured by bootstrap analysis based on 1,000 random replicates (Felsenstein, 1985).

### Whole genomic analyses

Whole genome sequencing was performed using PacBio HiFi long-read sequencing with a 20 kb SMRTbell library (Macrogen). The resultant long reads were de novo assembled using the Microbial Assembly Application in SMRT Link (v13.0). Whole genome sequence was annotated with the NCBI Prokaryotic Genome Annotation Pipeline (PGAP) (Tatusova et al., 2016) and metabolic pathways were inferred using BlastKOALA with reference to the KEGG pathway database (Kanehisa et al., 2016). In order to investigate degradation potential, a search for carbohydrate-active enzymes was performed with dbCAN3 (Zheng et al., 2023). For analyzing genomic relationship, *Flavobacterium* genomes available from the NCBI genome database were downloaded. To depict a genome-based phylogenetic tree, a set of 92 universal bacterial core genes were extracted from the *Flavobacterium* genomes using the up-to-date bacterial core gene set and pipeline (UBCG) (Na et al., 2018), and subjected to reconstruct a phylogenomic tree using FastTree (Price et al., 2009). The average nucleotide identity (ANI) values were calculated using ANI Calculator (Yoon et al., 2017). Digital DNA-DNA hybridization (dDDH) values were estimated using the GGDC website (http://ggdc.dsmz.de/distcalc2.php) (Meier-Kolthoff et al., 2022).

### Morphological, physiological, and chemotaxonomic analyses

The morphological characteristics and the presence of flagella were investigated using transmission electron microscopy (TEM, Hitachi) based on negative staining with 1% (v/v) uranyl acetate. Gram staining was determined using the Gram Stain kit (bioMérieux). Catalase and oxidase activities were determined by spraying 3% (v/v) H_2_O_2_ solution and Kovac’s reagent (bioMérieux) onto fresh colonies, respectively. Growth range and optimal temperature were evaluated at nine different temperatures (4, 10, 15, 20, 25, 30, 35, 37, and 45°C) in R2A broth (R2B; BD Diagnostics). Effect of pH was examined in R2B adjusted to pH 5.0–10.0 at increments of pH 1.0 intervals. The pH media were buffered using MES, MOPS, HEPES, and CHES to maintain pH values of 5.0, 6.0, 7.0–8.0, and 9.0–10.0, respectively. NaCl requirement was tested in R2B, followed by supplementing with 0–5% (w/v) at increments of 0.5% NaCl intervals. Cellular growth was determined by measuring optical cell density at 600 nm using a UV-Vis spectrophotometer (Optizen POP-V). Growth under anaerobic conditions was monitored using the AnaeroPackTM (Mitsubishi Gas Chemical) at 25°C up to two weeks. Hydrolysis of starch (1%, w/v), CM-cellulose (1%, w/v), and casein (3%, w/v) was investigated on R2A medium supplemented with these substrates. Hydrolysis of CM-cellulose was confirmed by drenching the colonies with 0.1% (w/v) Congo red solution and rinsing with 1 M NaCl. Degradation of DNA was investigated by clear zones surrounding colonies on DNase agar supplemented with methyl green (Kisan Bio). Other enzymatic and metabolic features of six *Flavobacterium* strains were evaluated through the application of API test systems (ZYM and 20NE, bioMérieux) and GENIII MicroPlates (BIOLOG) according to the manufacturer's instructions.

The cellular fatty acid profiles of six *Flavobacterium* strains were analyzed by gas chromatography (Agilent 7890B GC system). For fatty acid extraction, biomass was collected from colonies grown on the same sectors of R2A plates after incubating at 25°C until the mid-exponential phase was reached. The fatty acids were saponified, methylated, and extracted in accordance with the standard protocol of the MIDI (Microbial Identification System) (Sasser, 1990). The resulting fatty acid methyl esters were identified using the TSBA6 database of the MIDI (Sherlock ver. 6.2). The analysis of respiratory quinones and polar lipids was carried out using the analytical services of the Korean Culture Center of Microorganisms (KCCM). Respiratory quinones and polar lipids were extracted from freeze-dried cells according to the method described by Minnikin et al. (1984). The extracted quinones were purified using thin-layer chromatography and subsequently analyzed by high-performance liquid chromatography (HPLC) (Collins and Jones, 1981). Polar lipid extracts were separated using two-dimensional thin-layer chromatography (TLC) on 10 × 10 cm silica gel-coated plates (Merck). After developing in chloroform/methanol/water (65:25:3.8) and chloroform/acetic acid/methanol/water (40:7.5:6:1.8), functional group-containing lipids were identified by spraying the plates with solutions of molybdophosphate, ninhydrin, molybdenum blue, *α*-naphthol, and Dragendorf's reagent (Minnikin et al., 1977).

### Nucleotide sequence accession numbers

The 16S rRNA gene sequences of strains HNIBRBA15423^T^ and 25HG05S-40^T^ have been deposited in the GenBank/EMBL/DDBJ databases under accession numbers PZ097109 and PV555167, respectively. The complete genome sequences of HNIBRBA15423^T^ and 25HG05S-40^T^ are available under accession numbers CP199751 and CP199750, respectively.

## Results and Discussion

### Molecular phylogenetic analysis

The almost-complete 16S rRNA gene sequences of strains HNIBRBA15423^T^ (1,467 bp) and 25HG05S-40^T^ (1,463 bp) were deposited in GenBank. The 16S rRNA gene sequence of strain HNIBRBA15423^T^ showed the highest similarities to those of *Flavobacterium jejuense* EC11^T^ (98.5%), *F. profundi* TP390^T^ (97.2%), and *F. jumunjinense* HME7102^T^ (96.8%), whereas that of strain 25HG05S-40^T^ showed the highest similarities to those of *F. dankookense* ARSA-19^T^ (98.1%), *F. phycosphaerae* MK012^T^ (97.5%), and *F. buctense* T7^T^ (96.8%). The 16S rRNA gene sequence similarity between strains HNIBRBA15423^T^ and 25HG05S-40^T^ was 91.3%. In all 16S rRNA gene-based phylogenetic trees generated using the three inferring methods, strains HNIBRBA15423^T^ and 25HG05S-40^T^ were placed within a cluster comprising *Flavobacterium* species (Fig. 1). Strain HNIBRBA15423^T^ formed a robust subclade with *F. jejuense* and *F. profundi*, which was supported by 82–88% bootstrap values. However, Strain 25HG05S-40^T^ formed a loose subclade with *F. dankookense* and *F. phycosphaerae*, and its phylogenetic position was further resolved by genome-based phylogenetic analysis.

### Genome features

The genomes of strains HNIBRBA15423^T^ and 25HG05S-40^T^ were sequenced using the PacBio HiFi system with 232.0× and 308.0× in depth, respectively. After assembly, the complete genomes of strains HNIBRBA15423^T^ and 25HG05S-40^T^ were 4,299,722 bp and 3,455,619 bp in length, and were deposited at the GenBank database. CheckM analysis revealed high genome completeness and low contamination for both strains, with completeness and contamination estimates of 99.5% and 0.5% for strain HNIBRBA15423^T^, and 99.1% and 0.9% for strain 25HG05S-40^T^, respectively. The complete genomes of strains HNIBRBA15423^T^ and 25HG05S-40^T^ were each assembled into a single, closed circular chromosome with no gaps. The DNA G + C contents of the genomes were 29.7% and 36.6%, which are within the genus range (29.4–50.9%). Based on the PGAP results, 3,812 and 3,032 genes were predicted for strains HNIBRBA15423^T^ and 25HG05S-40^T^, respectively. These genomes included 3,731 and 2,965 protein-coding genes, 49 and 43 tRNA genes, and three copies of 16S rRNA genes for each, respectively (Table S1). The three copies of the 16S rRNA gene sequences on each genome were identical to their PCR-amplified counterparts.

The HNIBRBA15423^T^ and 25HG05S-40^T^ genomes commonly harbored diverse metabolic pathways such as gluconeogenesis, tricarboxylic acid (TCA) cycle, biotin synthesis, and ABC transporters of lipoproteins and lipopolysaccharides. Alpha-amylase (EC 3.2.1.1) was identified in both genomes, consistent with the starch-hydrolyzing activity observed in both strains. Metabolic pathway annotation revealed that the genome of strain HNIBRBA15423^T^ harbored genes associated with thiamine biosynthesis and nitric oxide (NO) and nitrous oxide (N₂O) reductases (*nor*BC and *nos*Z). Meanwhile, the 25HG05S-40^T^ genome harbored a set of genes encoding NADH dehydrogenase complex type I (*nuo*A-N) a highly energy-efficient respiratory complex (Archer and Elliott, 1995).

CAZyme annotation revealed differences in the carbohydrate-active enzyme profiles of strains HNIBRBA15423^T^ and 25HG05S-40^T^. Strain HNIBRBA15423^T^ was found to possess a total of 156 CAZymes, comprising 4 auxiliary activities (AAs), 20 carbohydrate-binding modules (CBMs), 8 carbohydrate esterases (CEs), 57 glycoside hydrolases (GHs), 62 glycosyltransferases (GTs), and 5 polysaccharide lyases (PLs). In comparison, strain 25HG05S-40^T^ contained 113 CAZymes, including 3 AAs, 7 CBMs, 6 CEs, 40 GHs, and 57 GTs, with no PLs detected. In accordance with the presence of diverse GHs in both strains, HNIBRBA15423^T^ and 25HG05S-40^T^ exhibited hydrolytic activity toward both starch and CM-cellulose. Further analysis of the GH complement identified genes belonging to glycoside hydrolase family 5 subfamily 7 (GH5_7; YAF06204.1 in HNIBRBA15423^T^ and YAE87798.1 in 25HG05S-40^T^) in both strains. As members of GH5_7 include enzymes capable of hydrolyzing *β*-1,4-linked glucans, the presence of these genes is consistent with the CM-cellulose-hydrolyzing activity observed in both strains. In addition, strain HNIBRBA15423^T^ harbored a gene assigned to glycoside hydrolase family 12 (GH12; YAF06279.1), a family that includes endo-*β*-1,4-glucanases involved in the hydrolysis of *β*-1,4-glucans. Thus, the phenotypic hydrolysis of starch and CM-cellulose, together with the identified CAZyme complement, further supports the carbohydrate-degrading potential of both strains. These distinct genomic characteristics reflect the metabolic diversity between the two novel strains.

In the phylogenomic trees, strains HNIBRBA15423^T^ and 25HG05S-40^T^ formed a robust clade with other *Flavobacterium* genomes (Fig. 2). Strain HNIBRBA15423^T^ formed a robust clade with *F. jejuense* EC11^T^ and the ANI and dDDH values between the two strains were 85.2% and 29.5%, respectively. Strain 25HG05S-40^T^ formed a robust clade with *F. wongokense* WG47^T^ and the ANI and dDDH values between the two strains were 78.6% and 21.5%, respectively (Table S3). These values were below the standard criteria values for species delineation of ANI (95–96%) and dDDH (70%) (Chun et al., 2018; Goris et al., 2007; Richter and Rosselló-Móra, 2009), indicating that strains HNIBRBA15423^T^ and 25HG05S-40^T^ represent novel species of the genus *Flavobacterium*.

### Morphological, physiological, and biochemical characterization

Strains HNIBRBA15423^T^ and 25HG05S-40^T^ were Gram-stain-negative and rod-shaped (Fig. S1). Both strains grew optimally at 25°C. Strain HNIBRBA15423^T^ grew at 4–30°C, pH 6.0–9.0 (optimum, pH 8.0), and with 0–5% NaCl (optimum, 1%). Strain 25HG05S-40^T^ grew at 10–35°C, pH 6.0–9.0 (optimum, pH 7.0), and with 0–1% NaCl (optimum, 0.5%). Both strains were positive for oxidase and catalase activities. No growth was observed under anaerobic conditions using AnaeroPack, indicating that both strains are aerobic. Marine strains, including HNIBRBA15423^T^, *F. jejuense* KCTC 42149^T^, and *F. profundi* KACC 18559^T^ showed 5% NaCl tolerance, whereas freshwater and soil strains 25HG05S-40^T^, *F. dankookense* KCTC 23179^T^, and *F. aquatile* KACC 11692^T^ exhibited lower NaCl tolerance. In terms of macromolecule hydrolysis, only strains HNIBRBA15423^T^ and *F. profundi* KACC 18559^T^ hydrolyzed DNA, starch, casein, and CM-cellulose. Other phenotypic characteristics are listed in Tables 1 and S1.

The major cellular fatty acids (> 10% of the total fatty acids) for both strains were iso-C_15:0_, iso-C_15:1_ G, and iso-C_17:0_ 3-OH. Additionally, strain 25HG05S-40^T^ contained iso-C_15:0_, iso-C_15:1_ G, iso-C_17:0_ 3-OH, and summed feature 9 (iso-C_17:1_ *ω*9*c* and/or 10-methyl C_16:0_) (Table 2). The fatty acid profiles of strains HNIBRBA15423^T^ and 25HG05S-40^T^ shared several major components with those of the related *Flavobacterium* type strains, such as iso-C_15:0_, iso-C_15:1_ G, and iso-C_17:0_ 3-OH. However, quantitative differences were observed among the strains including summed feature 9, C_15:1_ *ω*6*c*, and iso-C_16:0_. The predominant respiratory quinone for both novel strains was menaquinone-6 (MK-6). The polar lipids of strain HNIBRBA15423^T^ consisted of phosphatidylethanolamine (PE), four unidentified aminolipids, an unidentified aminophospholipid (APL), and ten unidentified lipids. The polar lipid profile of strain 25HG05S-40^T^ consisted of PE, four unidentified aminolipids, and two unidentified lipids (Fig. S2). These chemotaxonomic features, including iso-C_15:0_ as the major fatty acid, MK-6 as the sole respiratory quinone and PE as the major polar lipid, confirmed the affiliation of both strains to the genus *Flavobacterium*. Overall, strain HNIBRBA15423^T^ could be differentiated from *F. jejuense* by its ability to grow at 4°C, colony morphology, nitrate reduction, and starch hydrolysis. Strain 25HG05S-40^T^ could be differentiated from *F. dankookense* KCTC 23179^T^ and *F. wongokense* WG47^T^ by its growth temperature range, enzyme activities, and ability to hydrolyze starch and casein.

### Taxonomical conclusion

Strains HNIBRBA15423^T^ and 25HG05S-40^T^ could be clearly distinguished from their closest relatives by a number of phenotypic characteristics such as colony morphology, temperature range for growth, NaCl tolerance, enzyme activities, degradation capabilities including DNA and starch, and the compositions of cellular fatty acids. The 16S rRNA gene sequence similarities between strain HNIBRBA15423^T^ and *F. jejuense* (98.5%), and between strain 25HG05S-40^T^ and *F. dankookense* (98.3%) indicated their affiliation with the genus *Flavobacterium*. Furthermore, the low ANI (85.2% and 76.0%) and dDDH values (29.5% and 20.3%) between the two novel strains and their respective closest type strains were well below the widely accepted thresholds for species delineation. Taken together, the integrated phylogenetic, genomic, physiological, and chemotaxonomic data support that strains HNIBRBA15423^T^ and 25HG05S-40^T^ represent two distinct novel species of the genus *Flavobacterium*. Therefore, the names *Flavobacterium suaedicola* sp. nov. and *Flavobacterium wonjuense* sp. nov. are proposed, with HNIBRBA15423^T^ and 25HG05S-40^T^ as the type strains, respectively.

### Description of *Flavobacterium suaedicola* sp. nov.

*Flavobacterium suaedicola* (su.ae.di’co.la. N.L. fem. n. *Suaeda*, a botanical genus name; L. masc./fem. n. suff. -cola, dweller; N.L. masc./fem. n. suaedicola, a dweller of the plant *Suaeda*, referring to the isolation of the type strain from the halophyte *Suaeda maritima*).

Cells are Gram-stain-negative, aerobic, non-motile, and rod-shaped, approximately 2.2–4.9 µm in length and 0.3–0.4 µm in width. Colonies on R2A agar are yellow-pigmented, irregular, and convex. Growth occurs at 4–30°C (optimum, 25°C), at pH 6.0–9.0 (optimum, pH 8.0) and with 0–5% NaCl (optimum 1% NaCl). Oxidase- and catalase-activities are positive. In the API 20NE strip, esculin hydrolysis and gelatinase activity are positive, but nitrate reduction, indole production, glucose fermentation, arginine dihydrolase, urease, and *β*-galactosidase are negative. In the API ZYM strip, alkaline phosphatase, esterase (C4), esterase lipase (C8), leucine arylamidase, valine arylamidase, cystine arylamidase, trypsin, naphthol-AS-BI-phosphohydrolase, *α*-glucosidase, and *β*-glucosidase activities are positive; but lipase (C14), *α*-chymotrypsin, acid phosphatase, *α*-galactosidase, *β*-galactosidase, *β*-glucuronidase, *N*-acetyl-*β*-glucosaminidase, *α*-mannosidase, and *α*-fucosidase activities are negative. DNA, starch, casein, and CM-cellulose are hydrolyzed. The major cellular fatty acids (> 10%) are iso-C_15:0_, iso-C_15:1_ G, and iso-C_17:0_ 3-OH. The only respiratory quinone is MK-6. The polar lipids are phosphatidylethanolamine, four unidentified aminolipids, an unidentified aminophospholipid, and ten unidentified lipids.

The type strain is HNIBRBA15423^T^ (= HNIBR-BC8745^T^ = MCCC 1K10276^T^), isolated from the root of a halophyte associated with the halophyte *Suaeda maritima* in Taean, Republic of Korea. The length of the complete genome sequence of the type strain is 4.29 Mb with a DNA G + C content of 29.7%. GenBank accession numbers for the 16S rRNA gene sequence and the complete genome sequence of the type strain are PZ097109 and CP199751, respectively.

### Description of *Flavobacterium wonjuense* sp. nov.

*Flavobacterium wonjuense* (*won.ju.en’se*. N.L. neut. adj. *wonjuense*, of or pertaining to Wonju, a city in Gangwon Province, Republic of Korea, from where the type strain was isolated).

Cells are Gram-stain-negative, aerobic, non-motile, and rod-shaped, approximately 1.2–2.3 µm in length and 0.4–0.5 µm in width. Colonies on R2A agar are yellow-pigmented, convex, and circular with entire margins. Growth occurs at 10–35°C (optimum, 25°C), at pH 6.0–9.0 (optimum, pH 7.0), and with 0–1% NaCl (optimum 0.5% NaCl). Oxidase- and catalase-activities are positive. In the API 20NE strip, esculin hydrolysis is weakly positive, but nitrate reduction, indole production, glucose fermentation, arginine dihydrolase, urease, gelatinase, and *β*-galactosidase are negative. In the API ZYM strip, alkaline phosphatase, leucine arylamidase, valine arylamidase, and cystine arylamidase activities are positive; but esterase (C4), esterase lipase (C8), lipase (C14), trypsin, *α*-chymotrypsin, acid phosphatase, naphthol-AS-BI-phosphohydrolase, *α*-galactosidase, *β*-galactosidase, *β*-glucuronidase, *α*-glucosidase, *β*-glucosidase, *N*-acetyl-*β*-glucosaminidase, *α*-mannosidase, and *α*-fucosidase activities are negative. Starch, casein, and CM-cellulose are hydrolyzed, but DNA is negative. The major cellular fatty acids (> 10%) are iso-C_15:0_, iso-C_15:1_ G, iso-C_17:0_ 3-OH, and summed feature 9 (iso-C_17:1_ *ω*9*c* and/or 10-methyl C_16:0_). The only respiratory quinone is MK-6. The polar lipids are phosphatidylethanolamine, four unidentified aminolipids, and two unidentified lipids.

The type strain is 25HG05S-40^T^ (= FBCC-B19464^T^ = MCCC 1K10173^T^), isolated from a river in the Republic of Korea. The length of the complete genome sequence of the type strain is 3.45 Mb with a DNA G + C content of 36.6%. GenBank accession numbers for the 16S rRNA gene sequence and the complete genome sequence of the type strain are PV555167 and CP199750, respectively.

## Figures and Tables

**Fig. 1.**
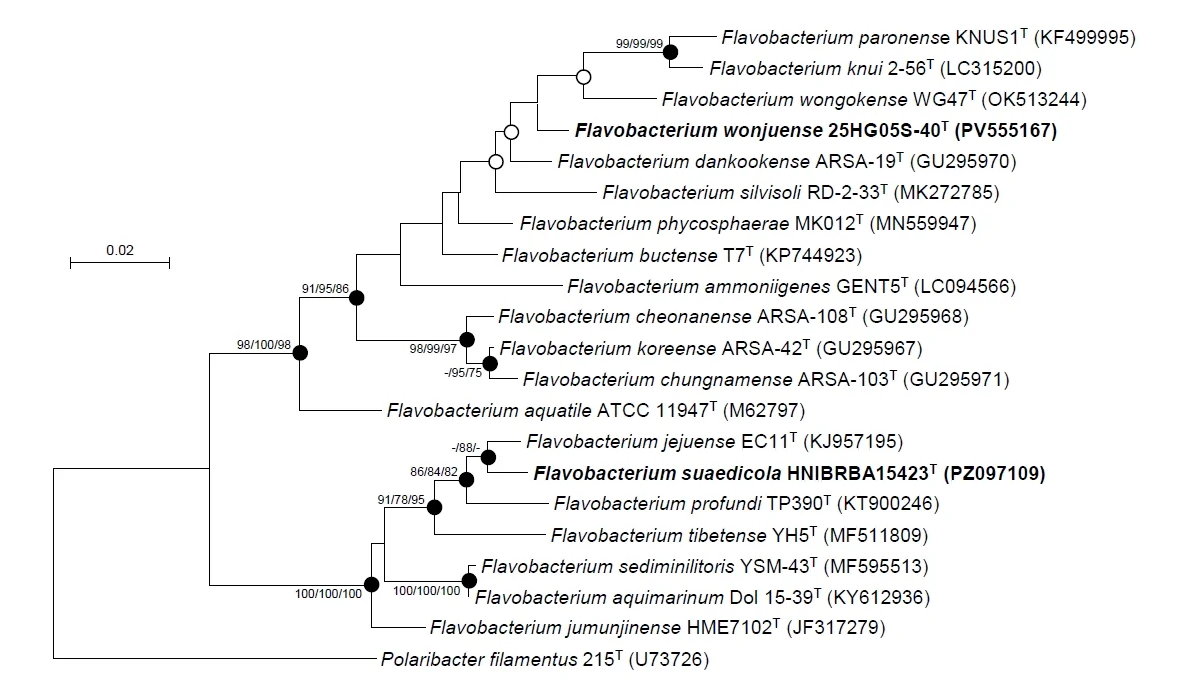
Phylogenetic tree showing the position of strains HNIBRBA15423^T^ and 25HG05S-40^T^. Maximum-likelihood phylogenetic tree based on almost full-length 16S rRNA gene sequences showing the relationships between strains HNIBRBA15423^T^, 25HG05S-40^T^, and the related type strains. Bootstrap values based on 1,000 replications over 70% are shown at nodes for maximum-likelihood, neighbor-joining and minimum-evolution methods, respectively. Filled circles indicate that the corresponding nodes were recovered by three methods. *Polaribacter filamentus* 215^T^ (U73726) was used as outgroup. Bar = 0.02 substitutions per nucleotide position.

**Fig. 2.**
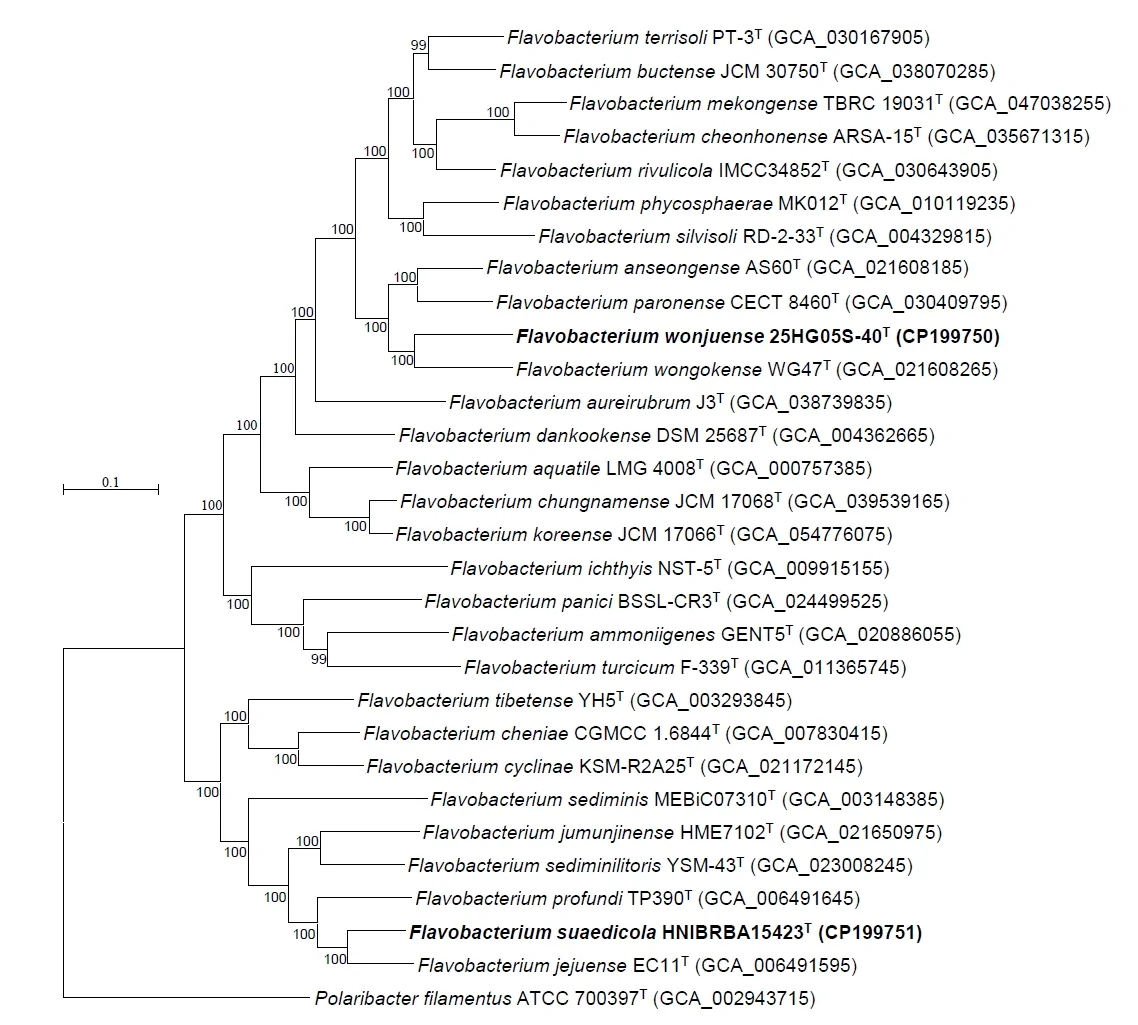
Phylogenomic tree including genomes of strains HNIBRBA15423^T^ and 25HG05S-40^T^, and other *Flavobacterium* species. The tree was reconstructed using the multiple-alignment of the 92 bacterial core genes. The genome sequence of *Polaribacter filamentus* ATCC 700397^T^ was used as an outgroup. Bootstrap values are shown at nodes. Bar = 0.1 substitutions per nucleotide position.

**Table 1.** Phenotypic characteristics of strains HNIBRBA15423^T^, 25HG05S-40^T^, and other *Flavobacterium* species Strains: 1, HNIBRBA15423^T^; 2, 25HG05S-40^T^; 3. *F. jejuense* KCTC 42149^T^; 4. *F. profundi* KACC 18559^T^; 5. *F. dankookense* KCTC 23179^T^; 6. *F. wongokense* WG47^T^, 7. *F. aquatile* KACC 11692^T^. Data are from this study unless otherwise indicated. All strains were positive for esculin hydrolysis, catalse, and oxidase. All strains were negative for activities of indole production, glucose fermentation, arginine dihydrolase, urease, *β*-galactosidase, lipase (C14), *α*-chymotrypsin, *α*-galactosidase, *β*-glucuronidase, *N*-acetyl-*β*-glucosaminidase, *α*-mannosidase, and *α*-fucosidase. +, positive; −, negative; A, aerobic; FA, facultatively anaerobic.

Characteristics	1	2	3	4	5	6^d^	7
Isolation source	root	soil	algae^a^	deep-sea sediment^b^	freshwater^c^	freshwater	freshwater^e^
Colony morphology	irregular	circular	circular^a^	circular^b^	circular^c^	circular	circular^e^
Temperature range (℃)	4–30	10–35	10–30^a^	4–35^b^	4–37^c^	10–30	10–30^c^
pH range	6.0–9.0	6.0–9.0	6.0–10.5^a^	6.0–9.0^b^	6.5–9.5^c^	6.5–9.5	6.5–8.0^f^
NaCl range (%)	0–5	0–1	0–5^a^	0–5^b^	0–1^c^	0–0.5	0–0.5^f^
NaCl optimum	1	0.5	1–2^a^	1^b^	0^c^	0	0^e^
Requirement for oxygen	A	A	A^a^	FA^b^	A^c^	A	A^e^
Activities of							
Nitrate reduction	-	-	+	-	-	-	-
Gelatinase	+	-	+	-	-	-	-
Esterase (C4)	+	-	+	+	+	+	+
Esterase lipase (C8)	+	-	+	+	+	+	+
Trypsin	+	-	+	+	-	-	-
Acid phosphatase	-	-	+	+	+	+	+
Naphthol-AS-BI-Phosphohydrolase	+	-	-	-	+	+	+
*α*-Glucosidase	+	-	+	+	-	-	+
*β*-Glucosidase	+	-	-	-	-	-	-
Macromolecule hydrolysis							
DNA	+	-	+	+	-	-	-
Starch	+	+	-	+	+	-	+
Casein	+	+	+	+	-	+	+
CM-cellulose	+	+	+	+	-	-	-

Data from

a Park et al. (2015);

b Wang et al. (2020);

c Lee et al. (2012);

d Jo et al. (2025);

e Webster and Hugh (1979);

f Jo et al. (2023).

**Table 2.** Cellular fatty acid composition of strains HNIBRBA15423^T^, 25HG05S-40^T^, and other *Flavobacterium* species Strains: 1, HNIBRBA15423^T^; 2, 25HG05S-40^T^; 3, *F. jejuense* KCTC 42149^T^; 4, *F. profundi* KACC 18559^T^; 5, *F. dankookense* KCTC 23179^T^; 6, *F. wongokense* WG47^T^; 7, *F. aquatile* KACC 11692^T^. All data were obtained in the present study except for those of *F. wongokense* WG47^T^. -, not detected; tr, < 1%.

Fatty acids	1	2	3	4	5	6^a^	7
Saturated							
C_16:0_	-	-	-	tr	tr	2.5	tr
Hydroxylated							
C_15:0_ 3-OH	-	-	-	-	-	-	3.9
C_16:0_ 3-OH	-	-	-	tr	-	4.5	-
C_17:0_ 2-OH	-	-	tr	-	1.3	tr	2.1
iso-C_15:0_ 3-OH	8.0	9.2	7.4	6.7	7.4	9.5	6.4
iso-C_16:0_ 3-OH	4.9	2.3	2.3	2.5	3.8	4.5	4.5
iso-C_17:0_ 3-OH	12.8	14.6	12.9	12.0	10.7	14.6	6.3
Branched							
iso-C_13:0_	tr	-	1.0	tr	-	-	-
iso-C_14:0_	1.3	-	1.5	1.5	1.0	tr	1.1
iso-C_15:0_	24.5	23.4	25.9	28.0	24.3	16.4	18.1
iso-C_15:1_ G	19.9	17.5	22.2	23.1	9.7	6.4	10.3
iso-C_16:0_	2.4	2.9	2.3	2.2	7.2	12.6	2.1
iso-C_16:1_ H	2.4	-	2.0	1.5	2.2	1.6	1.2
iso-C_17:0_					1.0	-	
anteiso-C_15:0_	2.0	2.1	1.6	tr	2.6	tr	6.3
anteiso-C_15:1_ A	tr	1.9	tr	-	tr	-	2.5
Unsaturated							
C_15:1_ *ω*6*c*	7.1	5.5	5.6	5.3	3.4	3.3	22.2
C_17:1_ *ω*6*c*	tr	4.6	tr	-	1.9	3.1	1.1
C_17:1_ *ω*8*c*	2.7	3.0	1.8	2.1	2.5	2.4	5.6
anteiso-C_17:1_ *ω*9*c*	-	-	-	-	1.6	-	-
Summed features^*^							
3	4.7	3.1	3.4	6.0	4.6	5.4	3.1
4	1.5	-	1.3	1.7	-	-	-
9	3.2	10.2	5.3	2.4	10.8	4.6	2.0

* Summed feature 3 comprises C_16:1_ *ω*7*c* and/or C_16:1_ *ω*6*c*, summed feature 4 comprises iso-C_17:1_ I and/or anteiso-C_17:1_ B, and summed feature 9 comprises iso-C_17:1_ *ω*9*c* and/or 10-methyl C_16:0_. Data from

a Jo et al. (2025). Cellular fatty acids of strain *F. wongokense* WG47^T^ were extracted from cells grown 25°C on R2A agar.

## Supplementary Information

Table S1. Genomic features of strains HNIBRBA15423^T^, 25HG05S-40^T^, *F. jejuense* EC11^T^, and *F. dankookense* DSM 25687^T^

Table S2. Pairwise genomic relatedness between strain HNIBRBA15423^T^, 25HG05S-40^T^, and closely related *Flavobacterium* species based on16S rRNA gene, and average nucleotide identity (ANI), and digital DNA–DNA hybridization (dDDH)

Table S3. Carbon source utilization patterns (GENIII MicroPlate) of the *Flavobacterium* strains Strains: 1, HNIBRBA15423^T^, 2, 25HG05S-40^T^, 3, *F. jejuense* KCTC 42149^T^, 4, *F. profundi* KACC 18559^T^, 5, *F. dankookense* KCTC 23179^T^, 6, *F. aquatile* KACC 11692^T^, All data were obtained in this study. +, positive; -, negative. All positive for D-maltose, *α*-D-glucose, D-mannose, gelatin, L-glutamic acid, and all negative for D-trehalose, D-turanose, stachyose, D-raffinose, *α*-D-lactose, D-melibiose, *β*-methyl-D-glucoside, D-salicin, *N*-acetyl-D-glucosamine, *N*-acetyl-*β*-D-mannosamine, *N*-acetyl-D-galactosamine, *N*-acetyl neuraminic acid, 3-methyl glucose, D-fucose, L-fucose, L-rhamnose, inosine, D-sorbitol, D-mannitol, D-arabitol, myo-Inositol, glycerol, D-fructose-6-phosphate, D-aspartic acid, D-serine, L-alanine, L-histidine, L-pyroglutamic acid, D-gluconic acid, D-glucuronic acid, mucic acid, quinic acid, D-saccharic acid, p-hydroxy-phenylacetic acid, methyl pyruvate, D-lactic acid methyl ester, L-lactic acid, citric acid, *α*-keto-glutaric acid, D-malic acid, γ-amino-butyric acid, *α*-hydroxy-butyric acid, *α*-keto-butyric acid, propionic acid, and formic acid.

Fig. S1. Transmission electron micrographs of strains HNIBRBA15423^T^ (A) and 25HG05S-40^T^ (B).

Fig. S2. Two -dimensional thin layer chromatographs of polar lipids of strains HNIBRBA15423^T^ (A) and 25HG05S-40^T^ (B).
