# Supplementary material for: Description of two novel species, Flavobacterium suaedicola sp. nov. and Flavobacterium wonjuense sp. nov
Source: J Microbiol. 2026 Oct 31;64(10):e2607006. doi: 10.71150/jm.2607006 (PMC13728754; doi:10.71150/jm.2607006)
Supplement: Supplementary file 2 [file jm-2607006-Supplementary-Fig-S2.pdf]

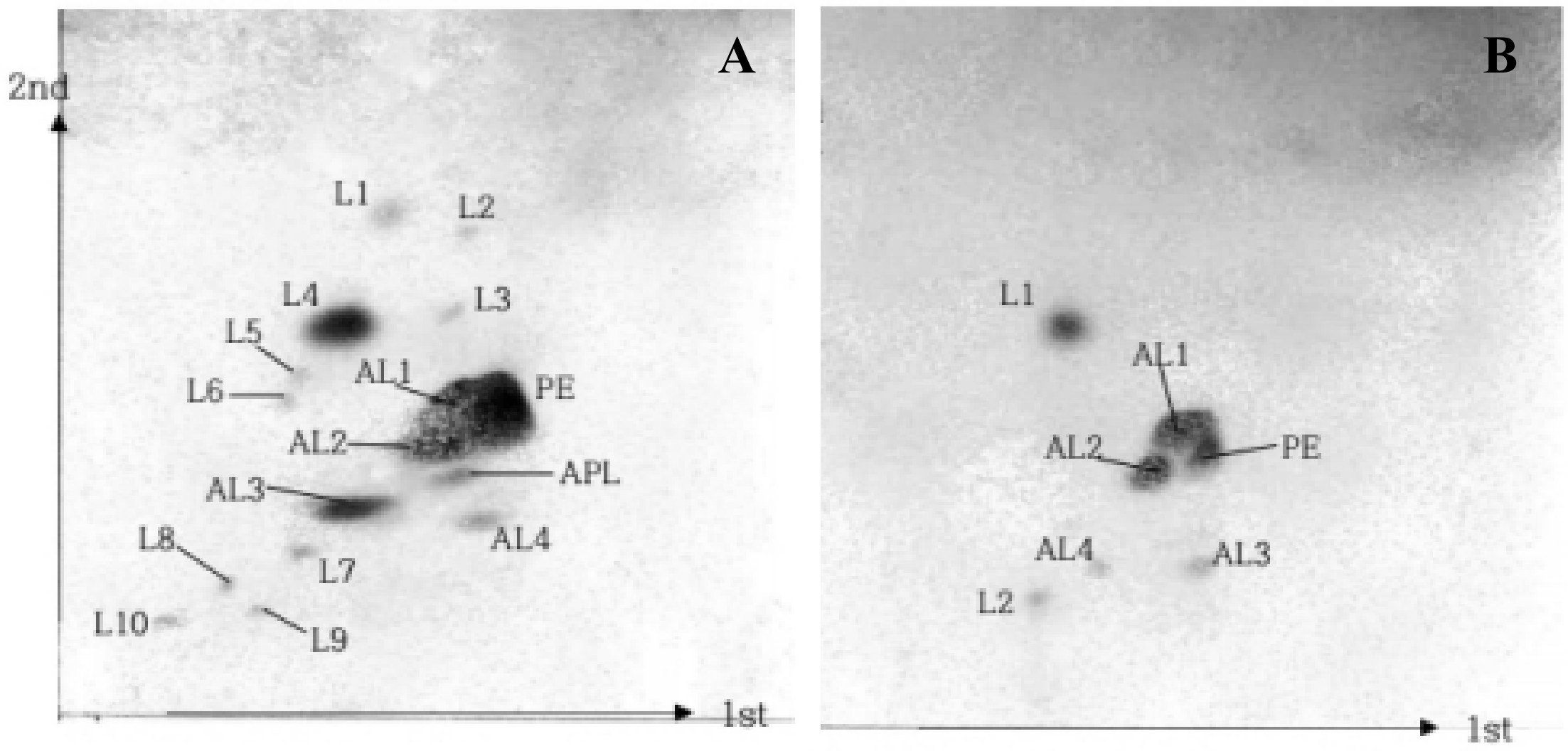

Fig. S2. Two-dimensional thin layer chromatographs of polar lipids of strains HNIBRBA15423^{T} (A) and 25HG05S-40^{T} (B).
