# Supplementary material for: Description of two novel species, Flavobacterium suaedicola sp. nov. and Flavobacterium wonjuense sp. nov
Source: J Microbiol. 2026 Oct 31;64(10):e2607006. doi: 10.71150/jm.2607006 (PMC13728754; doi:10.71150/jm.2607006)
Supplement: Supplementary file 3 [file jm-2607006-Supplementary-Table-S1.pdf]

Table S1. Genomic features of strains HNIBRBA15423^{T}, 25HG05S-40^{T}, *F. jejuense* EC11^{T}, and *F. dankookense* DSM 25687^{T}

Features	HNIBRBA15423^{T}	25HG05S-40^{T}	EC11^{T}	DSM 25687^{T}
Genome size (bp)	4,299,722	3,455,619	4,753,655	3,211,787
G + C %	29.7	36.6	29.9	32.4
No. Contigs	1	1	74	19
No. tRNA	49	43	47	36
No. rRNA	9	9	3	3
No. coding sequences	3,731	2,965	4,165	2,804
GenBank accession No.	CP199751	CP199750	VEVQ00000000	SNXR00000000
