# Supplementary material for: Description of two novel species, Flavobacterium suaedicola sp. nov. and Flavobacterium wonjuense sp. nov
Source: J Microbiol. 2026 Oct 31;64(10):e2607006. doi: 10.71150/jm.2607006 (PMC13728754; doi:10.71150/jm.2607006)
Supplement: Supplementary file 4 [file jm-2607006-Supplementary-Table-S2.pdf]

Table S2. Pairwise genomic relatedness between strain HNIBRBA15423^T, 25HG05S-40^T, and closely related *Flavobacterium* species based on16S rRNA gene, and average nucleotide identity (ANI), and digital DNA–DNA hybridization (dDDH)

Features	HNIBRBA15423^T (16S rRNA/ANI/dDDH)	25HG05S-40^T (16S rRNA/ANI/dDDH)
HNIBRBA15423^T	-	91.3/71.4/20.8
25HG05S-40^T	91.3/71.4/20.8	-
F. jejuense EC11^T	98.5/85.2/29.5	91.1/71.2/19.8
F. profundi TP390^T	97.2/80.2/23.0	91.4/71.2/20.2
F. tibetense YH5^T	96.2/76.0/20.4	91.1/73.1 /20.9
F. sediminilitoris YSM-43^T	96.6/79.8/22.6	91.8/73.3/20.1
F. dankookense DSM 25687^T	91.5/73.0/20.1	98.3/76.0/20.3
F. wongokense WG47^T	91.3/71.6/19.7	97.3/78.6/21.5
F. anseongense AS60^T	91.5/72.5/20.7	96.8/78.6/21.5
F. paronense CECT 8460^T	90.9/72.7/19.5	96.8/78.5/21.9
F. silvisoli RD-2-33^T	91.8/71.8/19.8	95.9/76.0/19.8
