# Supplementary material for: Description of two novel species, Flavobacterium suaedicola sp. nov. and Flavobacterium wonjuense sp. nov
Source: J Microbiol. 2026 Oct 31;64(10):e2607006. doi: 10.71150/jm.2607006 (PMC13728754; doi:10.71150/jm.2607006)
Supplement: Supplementary file 5 [file jm-2607006-Supplementary-Table-S3.pdf]

Table S3. Carbon source utilization patterns (GENIII MicroPlate) of the *Flavobacterium* strains

Strains: 1, HNIBRBA15423^{T}; 2, 25HG05S-40^{T}; 3, *F. jejuense* KCTC 42149^{T}; 4, *F. profundi* KACC 18559^{T}; 5, *F. dankookense* KCTC 23179^{T}; 6, *F. aquatile* KACC 11692^{T}. All data were obtained in this study. +, positive; -, negative. All positive for D-maltose, α- D-glucose, D-mannose, gelatin, L-glutamic acid, and all negative for D-trehalose, D-turanose, stachyose, D-raffinose, *α*-D-lactose, D-melibiose, *β*-methyl- D-glucoside, D-salicin, *N*-acetyl- D-glucosamine, *N*-acetyl-*β*- D-mannosamine, *N*-acetyl- D-galactosamine, *N*-acetyl neuraminic acid, 3-methyl glucose, D-fucose, L-fucose, L-rhamnose, inosine, D-sorbitol, D-mannitol, D-arabitol, *myo*-Inositol, glycerol, D-fructose-6-phosphate, D-aspartic acid, D-serine, L-alanine, L-histidine, L-pyroglutamic acid, D-gluconic acid, D-glucuronic acid, mucic acid, quinic acid, D-saccharic acid, *p*-hydroxy-phenylacetic acid, methyl pyruvate, D-lactic acid methyl ester, L-lactic acid, citric acid, *α*-keto-glutaric acid, D-malic acid, *γ*-amino-butyric acid, *α*-hydroxy-butyric acid, *α*-keto-butyric acid, propionic acid, and formic acid.

utilization of carbons	1	2	3	4	5	6
Dextrin	-	-	+	+	+	+
D-Cellobiose	+	+	+	-	-	-
Gentiobiose	-	-	+	+	-	-
Sucrose	+	+	-	-	+	+
D-Fructose	-	-	-	-	-	-
D-Galactose	-	+	-	-	+	+
D-Glucose-6-phosphate	-	-	+	-	-	+
Glycyl-L-proline	+	-	+	+	-	-
L-Arginine	-	-	+	-	-	-
L-Aspartic acid	-	-	+	-	+	+
L-Serine	-	-	+	+	-	-
Pectin	+	-	+	-	-	-
D-Galacturonic acid	+	-	+	+	-	-
L-Galactonic acid lactone	+	-	+	+	-	-
Glucuronamide	-	+	-	-	-	-
L-Malic acid	-	+	-	-	-	-
Bromo-succinic acid	-	-	-	-	-	-
Tween 40	+	-	+	-	+	+
β-Hydroxy-D, L-butyric acid	-	-	+	-	-	-
Acetoacetic acid	+	-	+	+	+	+
Acetic acid	+	-	+	+	+	+
